# A Holistic Approach to the Evaluation of the *Montado* Ecosystem Using Proximal Sensors

**DOI:** 10.3390/s18020570

**Published:** 2018-02-13

**Authors:** João Serrano, Shakib Shahidian, José Marques da Silva, Mário de Carvalho

**Affiliations:** 1 Rural Engineering Department, University of Évora, Instituto de Ciências Agrárias e Ambientais Mediterrânicas (ICAAM), Apartado 94, 7002-554 Évora, Portugal; shakib@uevora.pt (S.S.); jmsilva@uevora.pt (J.M.d.S.); mjc@uevora.pt (M.d.C.); 2 Agroinsider Lda. (University of Évora, spin-off), PITE, R. Circular Norte, NERE, Sala 18, 7005-841 Évora, Portugal

**Keywords:** animal tracking, *montado* ecosystem, pasture monitoring, proximal sensing

## Abstract

The *Montado* is a silvo-pastoral system characterized by open canopy woodlands with natural or cultivated grassland in the undercover and grazing animals. The aims of this study were to present several proximal sensors with potential to monitor relevant variables in the complex *montado* ecosystem and demonstrate their application in a case study designed to evaluate the effect of trees on the pasture. This work uses data collected between March and June 2016, at peak of dryland pasture production under typical Mediterranean conditions, in twenty four sampling points, half under tree canopy (UTC) and half outside tree canopy (OTC). Correlations were established between pasture biomass and capacitance measured by a commercial probe and between pasture quality and normalized difference vegetation index (NDVI) measured by a commercial active optical sensor. The interest of altimetric and apparent soil electrical conductivity maps as the first step in the implementation of precision agriculture projects was demonstrated. The use of proximal sensors to monitor soil moisture content, pasture photosynthetically active radiation and temperature helped to explain the influence of trees on pasture productivity and quality. The significant and strong correlations obtained between capacitance and pasture biomass and between NDVI and pasture nutritive value (in terms of crude protein, CP and neutral detergent fibre, NDF) can make an important contribution to determination of key components of pasture productivity and quality and implementation of site-specific pasture management. Animal tracking demonstrated its potential to be an important tool for understanding the interaction between various factors and components that interrelate in the *montado* ecosystem and to support grazing management decisions.

## 1. Introduction

### 1.1. Montado Ecosystem: Importance and Decline Signs

The *Montado* is a silvo-pastoral system characterized by open canopy woodlands of mainly *Quercus suber* and *Quercus rotundifolia*, mingled in some areas with other Mediterranean tree species, with natural or cultivated grassland in the undercover [1] and grazing animals [2]. Covering about 3.5 million ha in the South-East region of the Iberian Peninsula [3] it is broadly classified as a high nature value (HNV) system, since it corresponds to farmland hosting a high level of biodiversity [4]. Nevertheless, a decline of these ecosystems has been reported since the end of the nineteenth century in southern Portugal, which has intensified during the recent decades [5]. The decline in the total area, in the tree cover density and in biodiversity, has serious implications on the structural diversity which is crucial for the maintenance of *montado* HNV [4]. Consensual reports in the literature suggest that this decline is due to a number of factors, such as environmental constraints, forest diseases, inappropriate management, and socioeconomic issues [5]. According to Garrido et al. [2], this sharp decline is due to land use change, including both intensification and abandonment of agriculture. For example, livestock overgrazing has been contributing to a reduction in natural regeneration and biodiversity, whereby adaptive and efficient management systems are needed to avert these potential risks [6].

### 1.2. Complexity of Montado Evaluation

In the *montado*, monitoring of indicators is particularly complex due to the existence of different strata (soil, pasture, trees and animals). Although these strata are interrelated, they are nevertheless object of distinct management strategies and practices, and have distinct functional roles in the equilibrium of the system and the resulting goods and services [1].

In order to ensure their sustainable management it is imperative to produce knowledge on the resilience thresholds of these systems [1], which requires considerable interdisciplinary research to link the recent developments in precision agriculture (PA) technology with grassland science and with animal science [7].

The timely evaluation of soil and pasture variability is an important challenge. Traditional soil and crop sampling is based on low sample resolution data collected typically at one composite sample per 1–3 ha [8]. Though the traditional “clip-and-weigh” methods of measuring biomass are highly accurate, they are costly, destructive, labor-intensive and time-consuming when employed for obtaining biomass properties at a high sampling density [9]. The major limitation of management zones (MZ) delineation based on traditional laboratory analyses is the prohibitive cost of relying on a large number of samples needed to explore within-field variability at proper spatial or temporal scales [8].

Pastures are highly heterogeneous systems due to variations in sward structure, composition and phenology as well as continuous changes caused by different drivers such as environmental factors and grazing [9]. In addition to different vegetation types, the annual variations of floristic composition and vegetation dynamics introduce significant variability and uncertainty into standardized sensing techniques used in permanent grasslands. High seasonal fluctuations of light, temperature and soil moisture drive plant quality and availability [10]. Further, the situation in grassland becomes more complicated when grazing animals are involved. In contrast to equal defoliation of the sward for silage and hay, the grazing animals create specific spatial patterns of sward biomass that change over time with considerable effects on the spatial heterogeneity of the grassland field [7].

### 1.3. Contribution of Precision Agriculture to the Sustainability of the Montado 

At the core of PA is the effective management of spatial and temporal variability related to all aspects of agricultural production for the purpose of improving crop performance and environmental quality [11]. This in turn requires the availability of efficient and accurate techniques for measuring within-field variations in soil properties and crop development at a very fine spatial scale [12]. Assessing variability is the first critical step and a necessary condition in PA, therefore, any PA system must first address the availability of measurement technologies that allow the mapping and understanding of this variability. While technology can facilitate the application of PA, it is only knowledge and interpretation of variability that makes it feasible [11].

With recent advancements associated to PA, information technologies, remote and proximal sensing and geospatial analyses supported by global positioning systems, rapid and non-destructive methods are becoming available for mapping the spatio-temporal changes of soil and vegetation characteristics within fields with high spatial resolution [9]. New proximal sensors allow the collection of samples at a high spatial resolution (>1500–2000 readings per ha), enabling the exploration of the spatial variability of pastoral ecosystems at fine scale [8], making it possible to evaluate the variables that determine the equilibrium and sustainability of this ecosystem [8] and to support grazing management decisions [9].

On-site and timely information on soil and biomass and their spatial distribution is needed for site-specific pasture management, and can help livestock managers make critical decisions in terms of planning grazing time, grazing period, grazing interval, stocking rate and inputs such as fertilizers [13].

PA research can be understood as a framework for reducing decision uncertainty that is caused by uncontrolled spatial and temporal variation [7]. Research on PA would develop more rapidly in grasslands if adequate sensors were available that support rapid non-destructive measurement of sward and soil characteristics on-the-go. In other words, the question is what to measure, when, with what sensor and under what conditions? [7]. Usually, the first approach is based on soil and/or relief information, derived from topographic maps, direct soil sampling, non-invasive sampling of soil apparent electrical conductivity (EC_a_) measured by electromagnetic induction or electrical resistivity sensors, and crop canopy characteristics measured by proximal or remote sensing sensors [8,12]. Normalized difference vegetation index (NDVI) is the most common crop parameter used in MZ delineation, which can be derived from satellite, airplane, or unmanned aerial vehicle (UAV) imagery, or can be created using commercial proximal optical crop sensors such as Crop Circle, Yara N-Sensor, GreenSeeker [8] or OptRx.

Remote sensing, through satellite imagery, is an interesting prospect due to the scale of the response, speed of processing and low cost. Particularly hyper-spectral imaging has been found to be a promising non-destructive tool for estimating and mapping nutrient concentrations of vegetation with a very short turn-around time across large areas [14,15]. Satellite images with different geometric and spectral characteristics (i.e., “Landsat 8”, “Sentinel-1” or “Sentinel-2”) are examples with applications in the *montado* ecosystem. They provide data at a spatial resolution of 10 to 30 meters, capable of recognizing patterns relevant to cork oak management [16]. Edirisinghe et al. [17] developed a method for quantitative prediction and mapping of annual pastures biomass under grazing using NDVI derived from high-resolution satellite imagery. Sentinel-1 and Sentinel-2 satellites are also suitable for monitoring pasture development due to their temporal resolution (5 days). Applications of satellite observations are, however, often limited due to the coarse spatial resolution [18] associated with poor quality image on cloudy days [19]. In alternative to traditional satellite-based remote sensing, UAVs provide high-resolution images in real time for PA applications with the advantage of being more flexible and more independent of climatic variables for the extraction of agriculturally-useful information to predict several physiological variables [12,20,21].

In the *montado* ecosystems, satellite, airplane or UAV images have the disadvantage of not being able to access the pasture under the trees, which highlights the importance of proximal sensors as a solution for monitoring pasture under these conditions [19]. Advances in non-invasive techniques, based on proximal sensing technology and data analysis techniques provide information on soil, crops and associated environmental properties [12]. Monitoring progress over the production period and environmental states can be used in site-specific farming to tailor specific crop and soil treatments for each field [22]. From this perspective and according to Almeida et al. [4], grazing is a central activity determining the long-term sustainability of the *montado* system. Extensive livestock production is supported by natural and biodiverse pastures, characterized by marked seasonal variation of biomass, plant species and growth stage. The use of the food resources and the occupation of grazing space can be very heterogeneous in such conditions due to the ruminants’ grazing behaviour [6]. A better understanding of animal behaviour and environmental interactions are required to optimise the management of livestock and the spaces in which they graze [23]. Global navigation satellite system (GNSS) position loggers could determine livestock grazing preference and hence improve pasture management and paddock utilisation [23]. Combining technologies for monitoring spatial behaviour of livestock with technologies that monitor soil and pasture availability, offers potential solutions to emerging issues and the opportunity to improve the management and welfare of extensive animal production [23].

In this context, the aims of this study were to present several proximal sensors with potential to monitor relevant variables in the *montado* ecosystem and demonstrate their application in a case study.

## 2. Material and Methods

### 2.1. Site Characteristics

**Figure 1:**
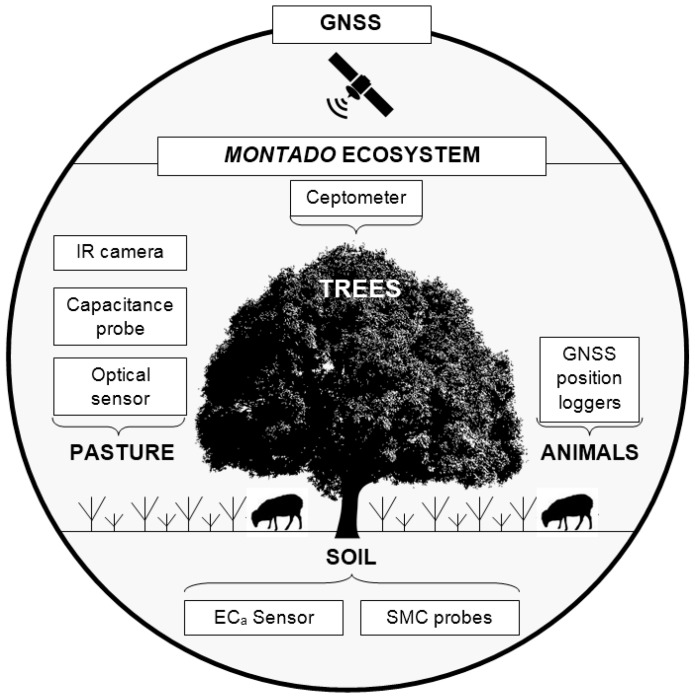
Proximal sensors used in this study for monitoring the *montado* ecosystem.

The studied field, with an area of 2.3 ha (Figure 1), is located at the Mitra farm (coordinates 38°32.2′ N; 8°01.1′ W), at Évora University, Southern Portugal. This field of oak trees (*Quercus ilex* ssp. *rotundifolia* Lam.), with a relatively reduced tree density (approximately 10 trees ha^−1^), has had an understory of natural pasture for the past thirty years and is grazed by sheep in a rotational system. A permanent bio-diverse pasture (legumes and grasses) was planted in October 2013, at the same time that 150 kg ha^−1^ of phosphate fertilizer (18% superphosphate) was applied. Since March 2014, the pasture has been grazed permanently by 15 adult Black Merino sheep.

The predominant soil of this field is classified as a Cambisol derived from granite [24]. Cambisols are characterized by slight or moderate weathering of parent material and by absence of appreciable quantities of illuviated clay, organic matter, aluminium and/or iron compounds. Acid Cambisols are not very fertile and are mainly used for mixed arable farming and as grazing and forest land. Cambisols in undulating or hilly terrain are planted to a variety of annual and perennial crops or are used as grazing land.

The Mediterranean climate can be considered as a transition between temperate and dry subtropical climates. It is characterized by summer drought, variable rainfall, and mild or moderately cold winters. The monthly average temperature is between 8 °C and 26 °C; minimum temperatures are close to 0 between December and February. The annual rainfall in the region is between 400 mm and 600 mm; rainfall occurs mainly between October and March and is practically non-existent during the summer.

### 2.2. Experimental Methodology

**Figure 2:**
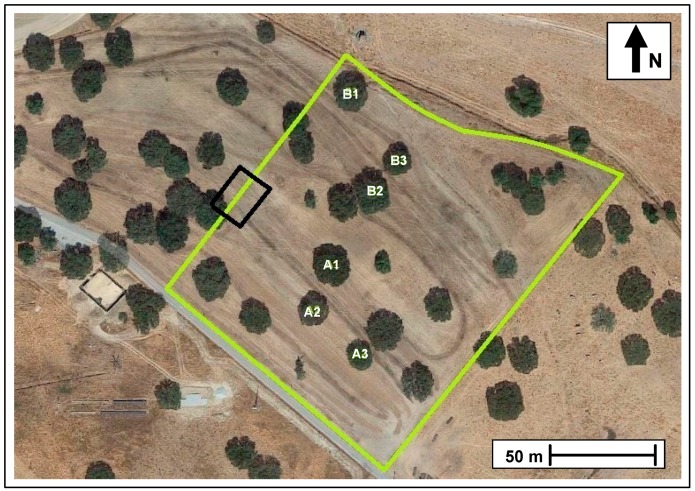
Experimental field (green line: outer boundary of the studied field; A1–A3 and B1–B3: tree codes).

The suitability of proximal sensors for monitoring relevant variables in the *montado* ecosystem (Figure 1) was demonstrated in a case study to measure the effect of trees on the pasture. In the study field (Figure 2) six trees were selected and twenty four sampling points were defined, twelve under tree canopy (UTC) and twelve outside tree canopy (OTC), half in the North (N) orientation and half in the South (S) orientation. The study was carried out between March and June 2016, which corresponds to the period of greatest vegetative development of dryland pastures in the Mediterranean region.

Correlations were established between pasture biomass and capacitance measured by a commercial probe, between pasture biomass and photosynthetically active radiation (PAR) measured by a ceptometer, and between pasture quality and normalized difference vegetation index (NDVI) measured by a commercial active optical sensor.

#### 2.2.1. Digital Elevation Survey

**Figure 3:**
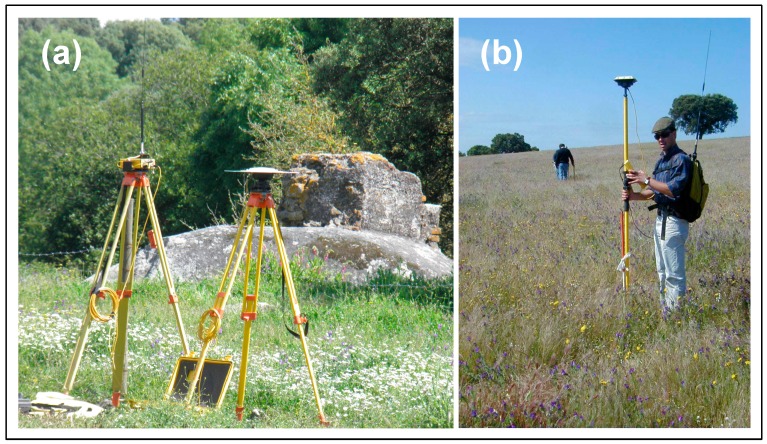
Trimble RTK/PP-4700 GPS: (**a**) station and radio; (**b**) rover.

A topographic survey of the experimental field was carried out using a real time kinematic (RTK) Global Positioning System (GPS) instrument (Trimble RTK/PP-4700 GPS, Trimble Navigation Limited, Sunnyvale, CA, USA, Figure 3). The altimetry data were sampled in the field on paths approximately 10 m apart. The digital elevation model surface was created using the linear interpolation triangulated irregular network (TIN) tool from ArcGIS 10.2 (Esri, Redlands, CA, USA) and converted to a grid surface with a 1 m grid resolution.

#### 2.2.2. Apparent Soil Electrical Conductivity (EC_a_) Survey

**Figure 4:**
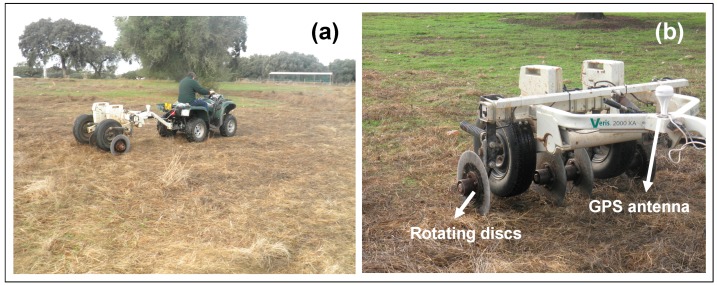
(**a**) Apparent soil electrical conductivity survey of the experimental field in October 2016; (**b**) Veris 2000 XA sensor.

The survey of apparent soil electrical conductivity (EC_a_) of the experimental field was carried out using a contact-type sensor (Veris 2000 XA, Veris Technologies, Salina, KS, USA, Figure 4) equipped with a GPS antenna. This soil resistance sensor is mounted on a chassis supported on two wheels and the actual sensor elements are formed by two pairs of coulter-electrodes (rotating discs). This sensor generates one set of topsoil data, weighted depth readings, representative of the 0 to 0.30 m layer. The sensor, pulled by an all-terrain vehicle, was programmed to register measurements every second. Further detail on the use of this sensor in monitoring soils in Mediterranean pastures can be found in the work carried by Serrano et al. [25].

#### 2.2.3. Soil Moisture Content (SMC) Measurements

**Figure 5:**
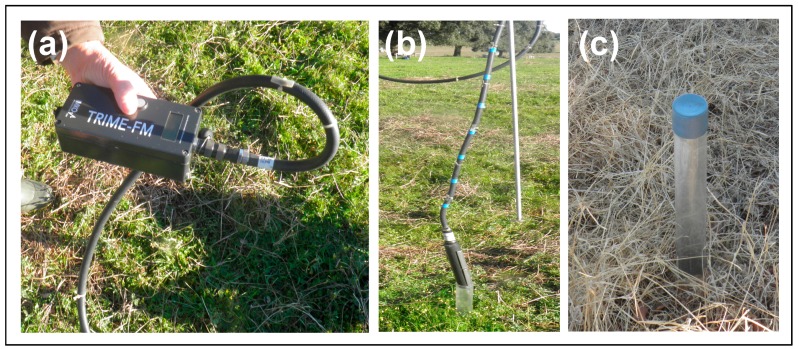
(**a**) TRIME-FM LC-display console; (**b**) TDR tube probe; (**c**) plastic tube installed in the ground.

Twenty four plastic tubes were installed in the ground at up to 0.60 m depth, twelve under tree canopy and twelve outside tree canopy. These probe access tubes permit rapid, reliable, and non-destructive recording of SMC profiles using portable Time Domain Reflectometry (TDR) probes (TRIME-FM, IMKO—Micromodultechnik, GmbH, Ettlingen, Germany, Figure 5). The measurements were carried out at the following depths: 0.10, 0.20, 0.30, 0.40, 0.50 and 0.60 m.

#### 2.2.4. Pasture Photosynthetically Active Radiation (PAR) Measurements

**Figure 6:**
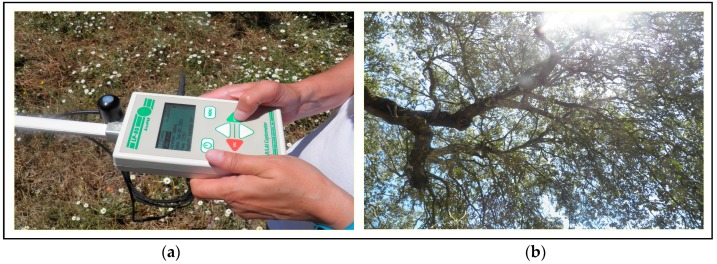
Ceptometer AccuPAR LP-80 (**a**) used to measure the photosynthetically active radiation (PAR) (**b**).

PAR is a crucial variable for modelling the vegetation development [26]. A ceptometer (AccuPAR LP-80, Decagon Devices, Pullman, WA, USA, Figure 6), composed of photosensors, was used to measure the radiation in the PAR spectral range (400–700 nm) of the twenty four sampling points at solar noon.

#### 2.2.5. Pasture Surface Temperature Measurements

**Figure 7:**
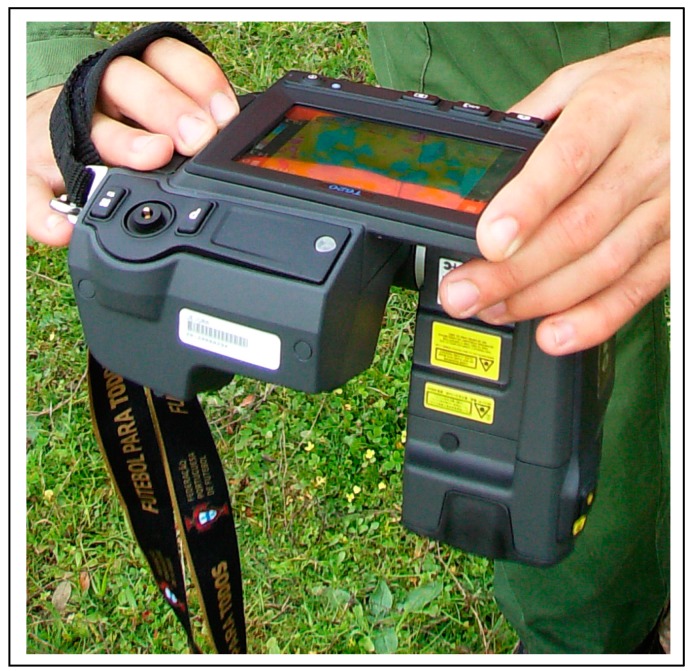
ThermaCAM^TM^ infrared camera used to measure the temperature at the pasture surface.

Infrared thermography is a technique that captures the emission of radiation by a body and converts it into a visible colored digital image. Infrared thermography images were obtained from each sampling point using an infrared camera (ThermaCAM^TM^ FLIR Systems, Wilsonville, OR, USA, Figure 7). Thermography images were analyzed using a ResearchIR^®^ 3.0 (FLIR Systems, Wilsonville, OR, USA) and data from each infrared image was exported to a spreadsheet where the information was processed in order to calculate the mean and standard deviation of pasture surface temperature.

#### 2.2.6. Pasture Biomass Estimation Using “Grassmaster II” Capacitance Probe

**Figure 8:**
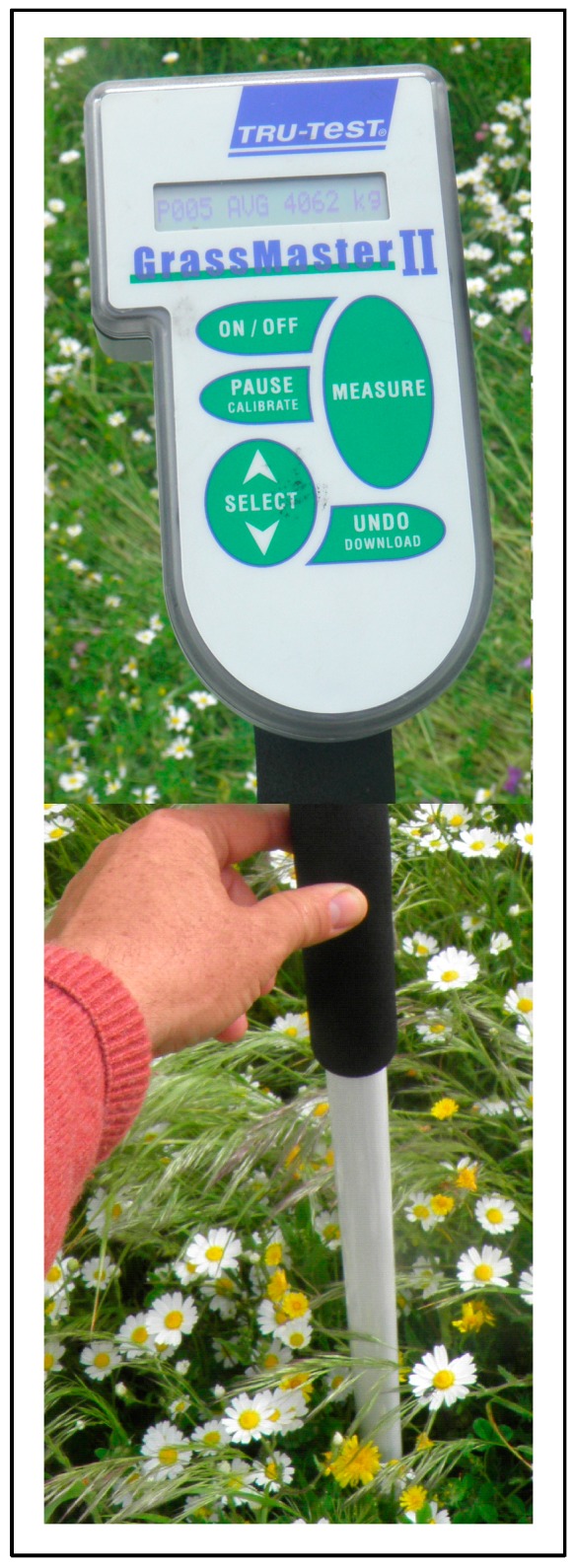
Grassmaster II electronic capacitance probe used to estimate pasture biomass.

The pasture biomass estimation was carried out by measuring all 24 sampling points with an electronic capacitance probe (Grassmaster II, Novel Ways Electronic, Hamilton, New Zealand, Figure 8), preceded by an air humidity level correction. The capacitance readings (CMR) were registered after the instrument had been positioned vertically over the vegetation, some 0.2–0.3 m away from the operator’s body. In each measuring area, ten readings were carried out with the probe and averaged. Further detail on the use of this sensor in monitoring Mediterranean pasture productivity can be found in the works carried out by Serrano et al. [27,28].

#### 2.2.7. Pasture Vegetation Index (NDVI) Measurement using “OptRx” Sensor

**Figure 9:**
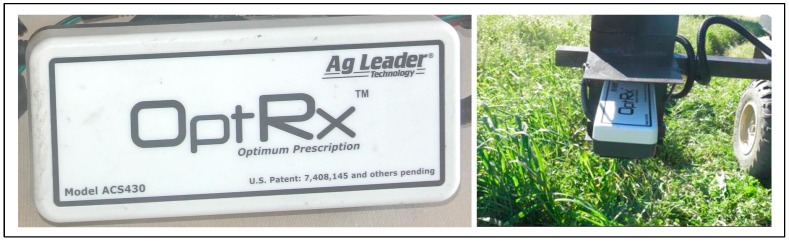
Active optical sensor “OptRx^TM^” used to measure NDVI.

The pasture vegetation index (NDVI) was measured in all 24 sampling points with an active optical sensor (AOS, OptRx^TM^, Ag Leader, Ames, IA, USA, Figure 9).

The sensor, placed on a platform standing 0.75 m above the ground (about 0.5 m above the pasture, considering an average pasture height of 0.25 m), measures simultaneously three visible and infrared bands: (i) RED (670 nm); (ii) RED EDGE (728 nm); and (iii) Near InfraRed (NIR, 775 nm). With two of the previous spectral bands NDVI vegetation index was calculated considering the following expression (Equation (1)):

(1) $$\mathrm{NDVI}=\frac{\mathrm{NIR}-\mathrm{RED}}{\mathrm{NIR}+\mathrm{RED}}$$

The operator stood still at the area of each geo-referenced point and performed measurements for a two minute period. The values of NDVI were organized in a spreadsheet and associated with the coordinates of the respective sampling points to calculate de mean and standard deviation of NDVI from about one hundred and twenty measurements taken at each point. Further detail on the use of this sensor in monitoring Mediterranean pasture quality can be found in the work carried out by Serrano et al. [27]. 

#### 2.2.8. Pasture Sample Collection and Analysis

Following the sensor readings, a metallic rim was placed on the ground and the pasture inside each sampling point was cut with a portable grass shears at 1–2 cm above ground level and stored in marked plastic bags. The pasture samples were then taken to the Animal Nutrition Laboratory of the University of Évora, where they were weighed, dried (for 72 h at 65 °C), and then weighed again to establish pasture productivity in terms of green matter (kg GM ha^−1^) and dry matter (kg DM ha^−1^) according to standard procedures [29]. The dehydrated samples were analysed in order to determine pasture crude protein (CP) and neutral detergent fibre (NDF) in % of DM [30].

#### 2.2.9. Animal Tracking with GPS Receivers

**Figure 10:**
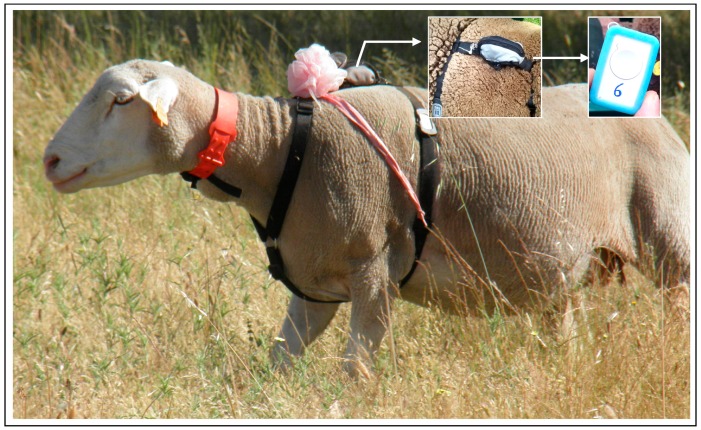
Animal GPS receiver “CatTrack^TM^” used to monitor the grazing patterns.

Six randomly selected ewes were fitted with GNSS position loggers (CatTrack^TM^, Perthold Engineering LLC, Anderson, Dallas, TX, USA, Figure 10) attached to harnesses. Loggers were programmed to record data every five minutes during a 24 h period in five consecutive weeks (days: 1, 8, 15, 22 and 29) of April 2016. Data were analysed using Trip@pc (Perthold Engineering LLC, Anderson, Dallas, TX, USA) to detect seasonal changes linked to the patterns of grazing location, pasture utilization and to manage grazing systems.

Further detail on the use of these sensors in monitoring sheep grazing can be found in the work carried by Sales-Baptista et al. [31].

## 3. Results and Discussion

To assess spatial variability at the very fine scale required by PA, different proximal sensors were used in this study.

### 3.1. Altimetry and EC_a_ Maps

**Figure 11:**
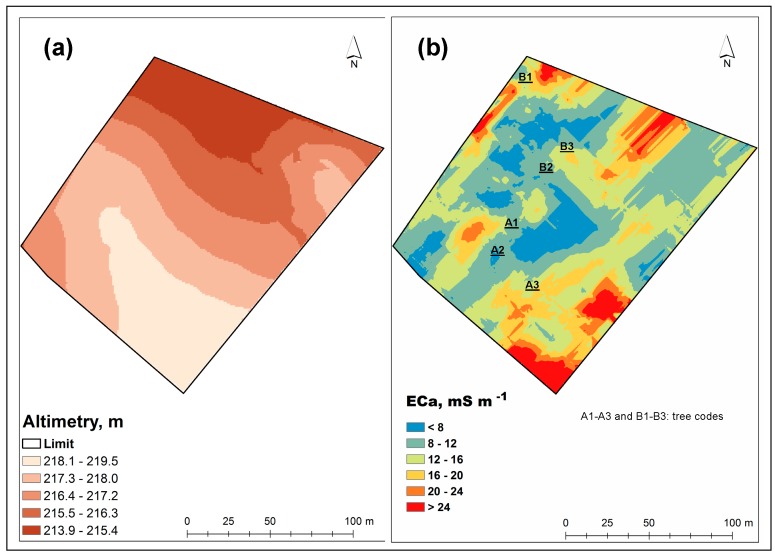
Altimetry map (**a**) and apparent soil electrical conductivity map (**b**) of the experimental field.

Figure 11 shows the maps of altimetry (a) and EC_a_ (b) of the experimental field. This spatial information obtained in an expeditious way with proximal sensors is usually the first step of precision agriculture projects [8]. Altimetric information is important inasmuch as it affects soil drainage and fertility and, in the case of the *montado* ecosystem, determines the animal comfort zones in the field throughout the year, depending on the air temperature and pasture availability. The spatial information of EC_a_ allows the definition of soil management zones, namely in terms of differentiated pasture fertilization [8].

**Figure 12:**
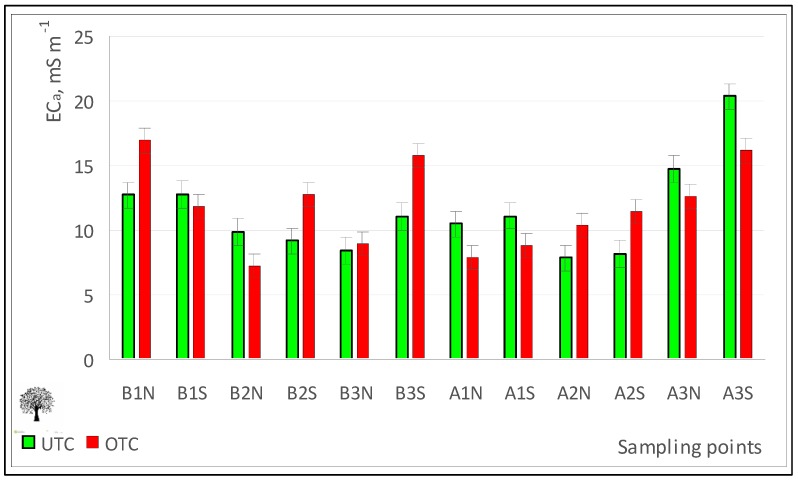
Apparent soil electrical conductivity (EC_a_) in April 2016, outside tree canopy (OTC) and under tree canopy (UTC).

In this particular case, the experimental field has a soft undulating topography, characteristic of this region, with only about 5 m of difference between the highest and the lowest sampled points (Figure 11a). On the other hand, the EC_a_ map (Figure 11b), shows a central zone of low EC_a_ (<8 mS m^−1^), while at the North and South ends of the field small patches with relatively high EC_a_ values (>24 mS m^−1^) emerge. This variability may indicate, for example, fertility, moisture or soil compaction issues and may in the future support smart soil sampling [32]. The combination of these surveys (altimetry and EC_a_) has a great potential for principal component analysis (PCA) in order to identify and select the factors that determine crop yield [32]. Regarding the sampling carried out at UTC and OTC, there were no significant differences in EC_a_ (Table 1; Figure 12).

### 3.2. Tree Influence on Pasture Productivity and Quality

**Figure 13:**
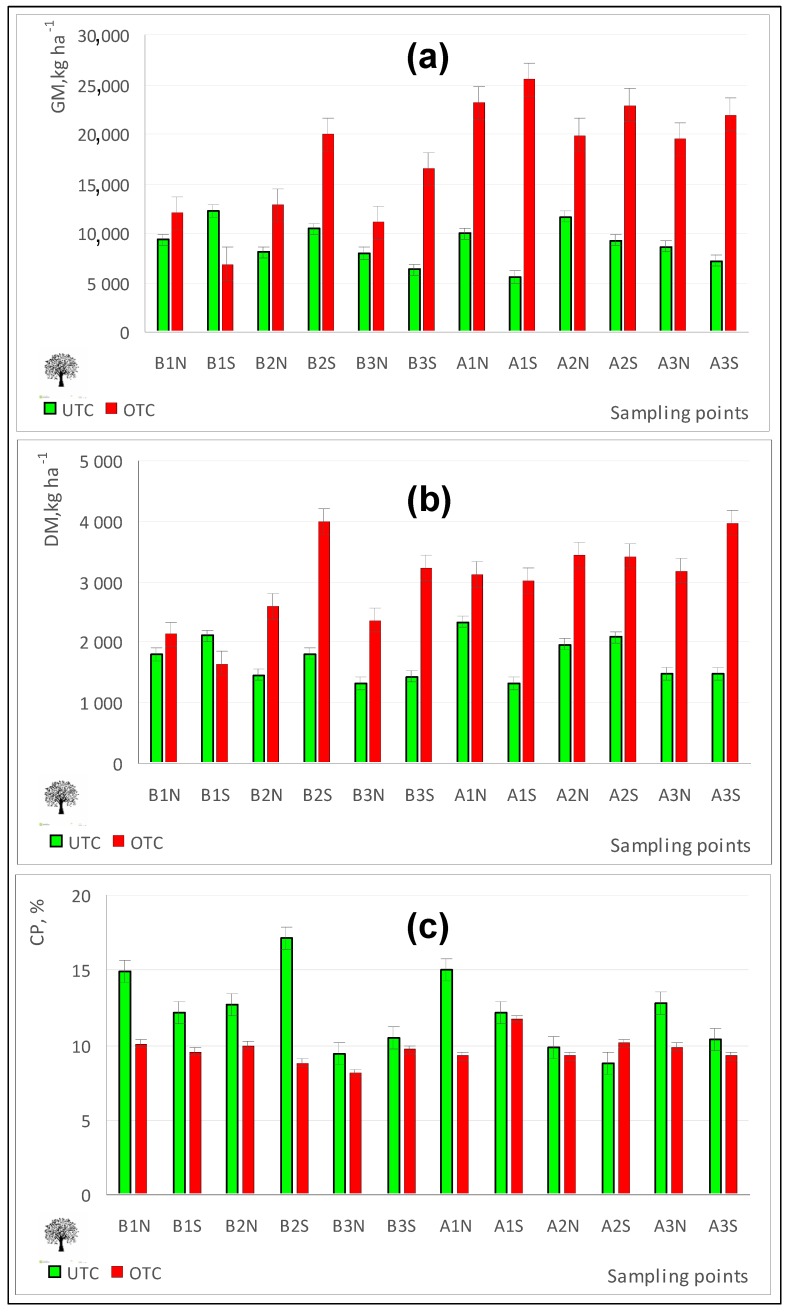
Pasture green matter, GM (**a**), dry matter, DM (**b**) and crude protein, CP (**c**) in April 2016, outside tree canopy (OTC) and under tree canopy (UTC).

The effect of trees on pasture is a direct consequence of the extent to which they modify the microclimate and soil properties [33,34]. Pasture production under tree canopy in silvo-pastoral systems normally depends on the degree of competition between trees and pasture for resources (light, moisture, and nutrients) [35]. At the peak of pasture production (April 2016), the results of this study showed (Figure 13a,b) significant differences in biomass production favouring the pasture productivity outside tree canopy (Table 1), which is in line with the study of Benavides et al. [34]. In addition to changes in the productivity, pastures in Mediterranean areas also exhibit changes in the quality of the forage due to the tree effect, changes which are related to the growth stage, nutrition, frequency and intensity of pasture use throughout the year and season [36]. In this study (Figure 13c), significant differences were found in pasture quality, with the tree canopy favourably influencing the pasture CP (Table 1), which confirms the results obtained by Pullanagari et al. [14]. Jackson and Ash [37] also showed a positive effect of trees on pasture quality and a negative effect on their productivity. 

### 3.3. Sensors Contribution to Understanding Tree Influence on Pasture Productivity and Quality.

Several authors have observed tree influence on pasture growth in the *montado* ecosystem. The most commonly mentioned factors are solar radiation, SMC and temperature. Benavides et al. [34] concluded that lack of light, cool temperatures and low SMC under trees reduce the growth rate of pasture species, and consequently delay their life cycle. Sousa et al. [38] attributed the higher quality of pasture under trees in terms of CP levels to the delay in the ontogenic development of shady plants (less advanced state of vegetative development), keeping them physiologically younger and allowing the maintenance of higher metabolic levels over a longer period of time. In this study SMC, PAR and temperature in April 2016 were significantly lower at UTC in comparison to OTC (Table 1).

#### 3.3.1. Soil Moisture Content Profiles

**Figure 14:**
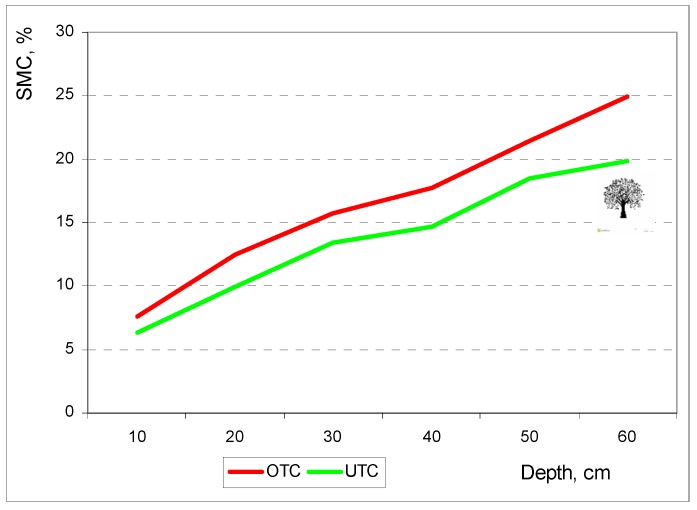
Soil moisture content (SMC) at different depths in April 2016, outside tree canopy (OTC) and under tree canopy (UTC).

Hussain et al. [35] showed that during spring shade has greater influence on pasture growth than soil moisture. The competition for water is usually the other main limiting factor of pasture growth, particularly in regions subjected to summer droughts with high recorded temperatures and incident radiation [34], as is the case of the southern region of Portugal. Figure 14 shows a soil profile of 0 to 0.60 m depth, showing lower SMC levels at UTC in all depths. 

A number of studies show that soil beneath trees has lower water content than soil beneath open pasture [34]. Additionally less rain falls on the understorey because some of it is intercepted by tree canopy and partly evaporates [34]. Soil water deficit results in lower forage dry matter yield, primarily due to limited leaf area development, and reduced photosynthesis caused by stomatal closure [34].

#### 3.3.2. Effect of Photosynthetically Active Radiation (PAR) on Pasture Productivity

**Figure 15:**
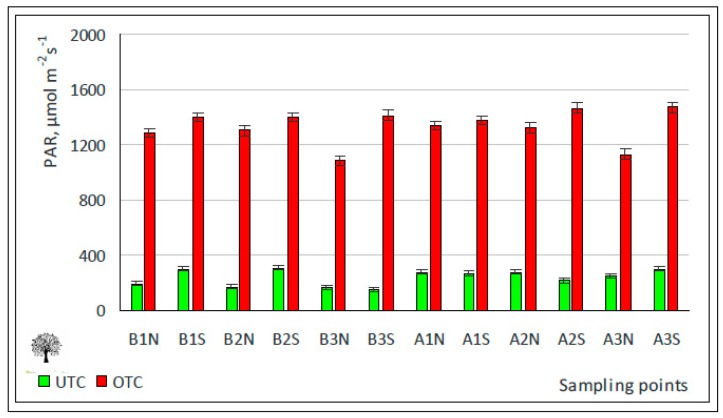
Photosynthetically active radiation (PAR) in April 2016, outside tree canopy (OTC) and under tree canopy (UTC).

Of all the competitive constraints, the level of shade and its duration (and, consequently, the interception of light) are among the most significant factors exerting negative effect on pasture production under trees [33,34,35,39] given that holm oak is an evergreen tree with a dense canopy [40]. The mean LAI of the six trees selected for this study was 4.42 ± 0.61 m^2^ m^−2^ and the mean value of canopy closure was 82%. Figure 15 shows the expected effect of tree canopy on the decrease of PAR that reaches the pasture, with systematically and significantly lower values of PAR under tree canopy (Table 1).

**Figure 16:**
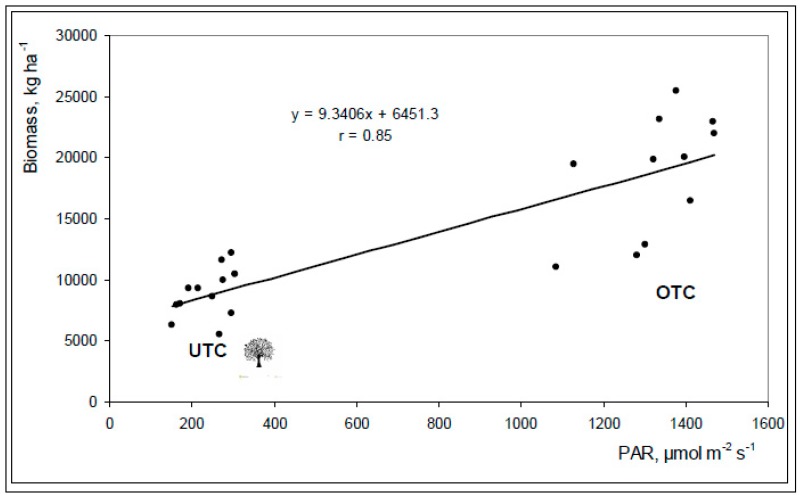
Relationship between photosynthetically active radiation (PAR) measured by ceptometer and pasture biomass productivity in April 2016, in all of the twenty four sampling points (under tree canopy, UTC and outside tree canopy, OTC).

Figure 16 shows the positive and significant correlation between PAR and pasture biomass production (*r* = 0.85; *p* < 0.01). 

This relationship between pasture productivity and light transmission to the understory is a consequence of the reduction of solar radiation and thus photosynthesis [41] and has been recorded by Guevara-Escobar et al. [33] and Benavides et al. [34]. A reduction in the quantity and quality of light directly affects the physiological processes of plants, decreasing pasture carbohydrate manufacture and net dry matter production [34].

#### 3.3.3. Pasture Surface Temperature Measurements

**Figure 17:**
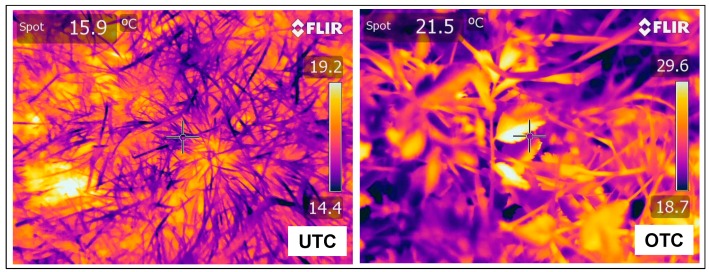
Thermal images obtained by infrared camera in April 2016, at two sampling points: one under tree canopy (UTC) and other outside tree canopy (OTC).

**Figure 18:**
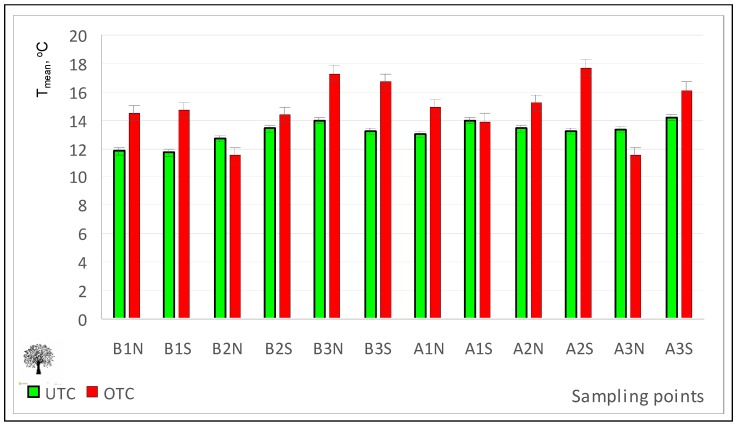
Mean temperature (T_mean_) in April 2016, outside tree canopy (OTC) and under tree canopy (UTC).

Pasture surface temperature measured with an infrared camera may be an indicator of the differential conditions of pasture development under or outside the canopy [33]. The infrared thermography images (Figure 17) allow the calculation of the mean temperature at pasture surface (Figure 18), a parameter that can help to explain the tree effect on pasture development. According to Benavides et al. [34] temperature is an important factor affecting pasture production because it affects the physiological processes of plants such as photosynthesis, respiration, and germination. Tree shade influences the microclimate of the crop, with daily and seasonal air and soil temperature variation decreasing with the proximity of the trees, as shown in this study (Figure 18; Table 1). This should have a direct consequence on the pasture phenology and productivity [40].

#### 3.3.4. Proximal Sensor for Estimating Pasture Productivity

**Figure 19:**
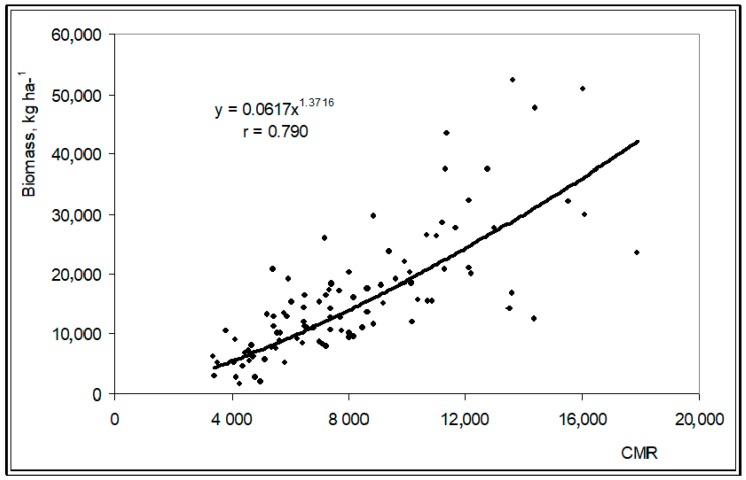
Relationship between capacitance (CMR) measured by the Grassmaster II probe and pasture biomass productivity between March and June2016, in all the twenty four sampling points.

The conventional methods for determining key components of pasture productivity and quality are time consuming and expensive [14], hence the interest in evaluating faster and cost effective tools. Measurements obtained by the Grassmaster II capacitance probe at the twenty four sampling points between March and June 2016 showed a significant correlation with pasture biomass (Figure 19; *r* = 0.79; *p* < 0.01).

These results confirm the practical interest of the Grassmaster II capacitance probe as a fast method of estimating the productivity of the Mediterranean pastures in Southern Portugal and are in line with the studies of Serrano et al. [27,28,29]. This is a fundamental step for decision making regarding the implementation of site-specific pasture management in terms of animal grazing or soil fertilizer application [27].

#### 3.3.5. Proximal Sensor for Estimating Pasture Quality

It is accepted that high CP and low NDF values reflect higher quality pastures [42]. Monitoring pasture nutritional value over time is critical for defining the nutritional value of pastures and designing balanced diets for grazing animals [36]. Optical sensing techniques have the potential to detect physiological and biochemical changes in plant ecosystems and according to Albayrak [43] canopy reflectance can be used for non-destructive prediction of forage quality variables in pastures.

**Figure 20:**
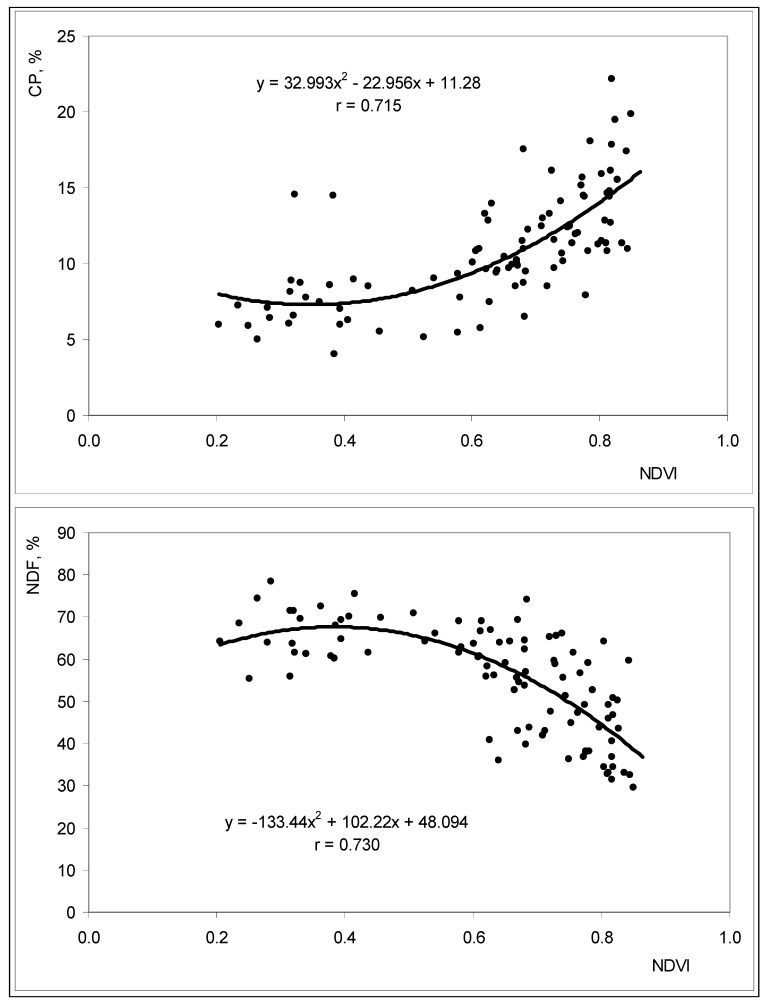
Relationship between NDVI measured by active optical sensor “OptRx^TM^” and pasture crude protein (CP) and neutral detergent fibre (NDF) between March and June 2016, in all the twenty four sampling points.

The OptRx^TM^ sensor detects vegetation with higher levels of chlorophyll (photosynthetically active vegetation), and this can be correlated with CP levels. Figure 20 shows the significant correlation between NDVI and CP (*r* = 0.72; *p* < 0.01) and NDVI and NDF (*r* = 0.73; *p* < 0.01), between March and June 2016. The correlation coefficients are of the order of magnitude of those obtained by Zhao et al. [44] (0.79 and 0.76, respectively for CP and NDF) with reflectance data obtained by remote sensing. Pullanagari et al. [14] also found satisfactory relationships between spectral measurements and pasture quality parameters such as CP and NDF contents, which can be attributed to absorbance of visible radiance by chlorophyll, abundant in green vegetation.

**Figure 21:**
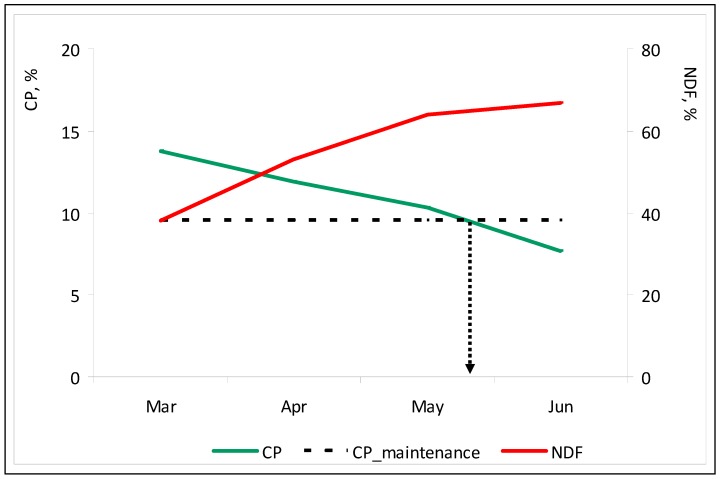
Evolution between March and June 2016 of the pasture nutritive value parameters (crude protein, CP, and neutral detergent fibre, NDF) in all the twenty four sampling points. The arrow indicates the sheep maintenance requirements in terms of CP.

This correlation can be important for the management of animal grazing intensity and calculation of food supplementation needs throughout the vegetative cycle of the pasture. Figure 21 shows the evolution of the pasture nutritive value parameters (CP and NDF), between March and June 2016. These results are in agreement with the typical patterns of pasture quality degradation in Mediterranean agro-silvo-pastoral systems during the vegetative cycle: reduction of CP and a continuous increase of NDF with the approach of the end of spring [10]. A critical moment occurs when CP content falls below the animal maintenance requirements −9.4% in sheep [45].

#### 3.3.6. Animal Tracking

Hussain et al. [35] concluded that the frequency of sheep grazing is also responsible for differences in pasture production and quality under trees relative to open pastures. Gómez-Rey et al. [46], for example, reported that soils under tree canopy present higher density and lower porosity as a result of the greater compaction caused by the animals. This situation may be responsible for a greater difficulty in root development which, together with the lower incidence of light, may explain the reduced development of the plants and differentiated adaptation of botanical species.

**Figure 22:**
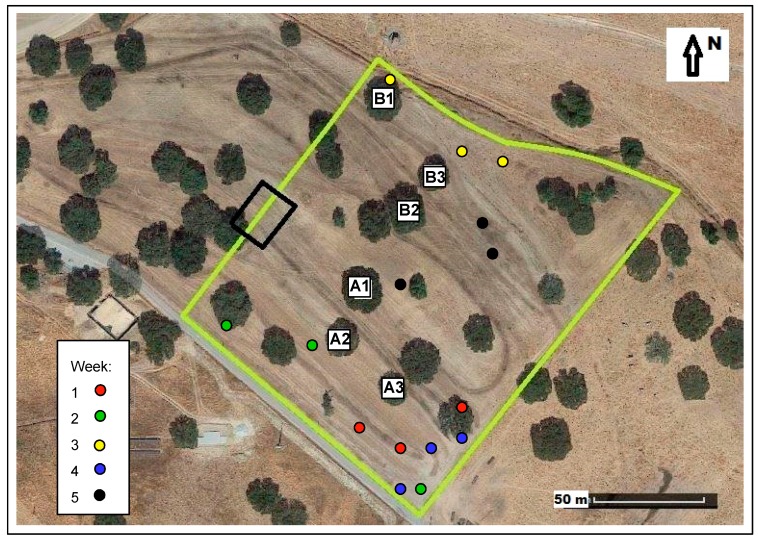
Location of the three most grazed patches in the studied field within a 24 h period in weeks 1 to 5 of April 2016 (green line: outer boundary of the studied field; A1–A3 and B1–B3: tree codes).

Figure 22 shows the location of three most grazed patches in the studied field within a 24 h period in the weeks 1 to 5 of April 2016. Although grazing occurred essentially at the Southern part of the paddock, corresponding to a greater predominance of legumes, it was observed that during these five weeks, corresponding to the beginning of spring, the animals searched several grazing sites. In a multi-diverse pasture, different plant species mature at different rates, driving animals to modify their grazing location according to the predominance of plant species. Grazing animals typically select sites where they can maximize the intake rate of energy and protein, which is correlated with biomass and quality [47]. Successful grazing and pasture management requires an understanding of the adjustment mechanism behind the grazing behaviour that enables adaptation to grazing conditions [6]. GPS receivers similar to the ones used in this study can become an important support tool for making decisions that are essential for a more precise pasture management [6] and for understanding the interaction between the various factors and components that interrelate in the *montado* ecosystem.

#### 3.3.7. Contribution of This Study to Montado Ecosystem Management

The “Precision Grazing/Precision Agriculture” research team of the University of Évora has been studying the *montado* since 2004, within the framework of a set of projects known as as (“Evaluation of technologies for monitoring the *montado* ecosystem: soil, plants and animals” (ECO-SPA). The main objective of this research is to produce knowledge in the PA field with potential for use by agricultural enterprises to support–semi or automatic–decision making in improving the *montado’s* ecosystem management. As such, various works have been published in the area of technologies for survey of soil and pasture spatial and temporal variability [27,28,48,49,50,51], for monitoring animal grazing [31], for evaluation of the tree effect on the soil and pasture [52], and for the differential application of fertilizers [53].

**Figure 23:**
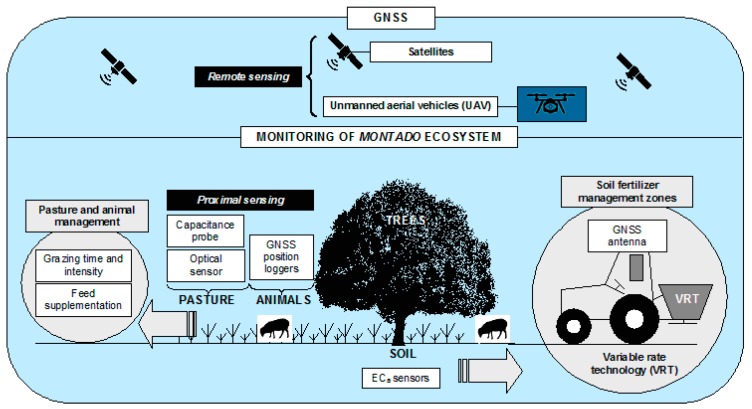
Global overview of technology contribution to *montado* ecosystem management.

The purpose of this article is to integrate knowledge and provide a concrete proposal for the *montado*. Figure 23 shows, on one hand, a global overview of this ecosystem, and on the other, the practical compromise which is desired for a methodology based on technology and the participation of small service providers which allow the implementation of the PA concept. More than trying to merge the sensors or develop mutisensor platforms, the purpose is to develop a framework that provides multiple answers at the level of the various components of the soil-pasture-tree-animal system that can impact its sustainability, and maintain it technically and economically competitive.

Today, the survey of the soil EC_a_ with contact or electromagnetic induction sensors can be carried out by specialized service providers at a reasonable price (of around 30 Euros ha^−1^). Given the possibilities provided in terms of identification of differential management zones and the subsequent intelligent soil sampling for the differential application of fertilizers, the use of these proximal sensors is a preliminary step in the application of site-specific management.

The significant correlations obtained in this work between NDVI (measured by the “OptRx”, AOS proximal sensor) and the pasture quality (in terms of CP and NDF) reveals the great potential of this index for monitoring the evolution of pastures along their growth cycle. Based on this data, alert maps for large pasture areas in the *montado* ecosystem can be generated, ensuring feed supplement at the times when the quality of the pasture is not satisfactory for the maintenance of the grazing animals.

Various authors have demonstrated the difficulty in expressing pasture productivity using NDVI, especially due to saturation which can occur at highly productive stages (such as peak spring production) [54]. As a result, various solutions have been proposed based on the use of multiple sensors (sensor fusion) which associate NDVI with other parameters that reflect the development of the pasture in terms of height [54]. This study has shown the suitability of the Grassmaster II capacitance probe for estimating pasture productivity, and thus the possibility of using it, especially during the spring, as a tool for fine tuning the calibration of the estimate obtained by NDVI. The knowledge of the pasture productivity allows the balanced management of animal density and rotation in the different fields.

Monitoring grazing animals provides a fundamental layer of information for the preservation of the ecosystem equilibrium, given the dynamic influence of the animals in terms of selective grazing and return of nutrients through dung and urine [7,55]. Today, the decrease in the cost of commercial animal GNSS receivers allows their wide spread use, with associated benefits in terms of animal wellbeing and safety.

Table 2 summarizes the main characteristics and applications of different sensing technologies that can be deployed in the study and management the *montado* ecosystem. From a scientific point of view, all the sensors used and trialed in the field are highly valuable in terms of producing knowledge for the operational and differential management of this ecosystem. Nevertheless, from the point of view of its applicability by the farm manager, remote sensing based in satellite imagery (“Sentinel-1” and “Sentinel-2) seems to be the most suitable, due to its low cost and intrinsic characteristics. Thus, during the next few years, the great challenge in terms of creation of knowledge applied to the management of *montado* ecosystem, will be to use the more expensive Technologies (soil electric conductivity sensors, proximal multispectral sensors, capacitance probes, animal GNSS receivers, etc.) to model and validate methods based on remote sensing. These should be able to monitor, detect, forecast and alert about the *montado* ecosystem, ensuring its balanced and sustainable management.

## 4. Conclusions

The *Montado* is a silvo-pastoral system constituted by multiple strata: soil, pasture, trees and animals. In the southern region of Portugal, the *montado* usually occupies poor soils, and is subject to characteristic Mediterranean restrictions in terms of temperature (very high) and rainfall (very low) from the end of the spring and throughout the summer, which cause low pasture productivity and justify low animal densities. In order to ensure sustainable management there is an urgent need to produce knowledge on the resilience threshold of these systems.

This study presents several proximal sensors with potential to monitor relevant variables and the relationships between the different strata in the *montado* ecosystem. Altimetry, apparent soil electrical conductivity, soil moisture content, photosynthetically active radiation, temperature and grazing data were measured to explain the influence of trees on pasture productivity and quality. Capacitance measured by Grassmaster II probes and NDVI measured by OptRx^TM^ active optical sensors show significant and strong correlation with pasture biomass and pasture crude protein, respectively, which demonstrates the practical interest of these proximal sensors from a precision agriculture perspective. This step is fundamental to help livestock managers make critical decisions in terms of planning grazing time and intensity, stocking rate, animal supplementation or soil fertilization.

## Figures and Tables

**Table 1:** Mean and standard deviation of measured parameters in April 2016 and probability of significant differences between under tree canopy (UTC) and outside tree canopy (OTC).

Parameter	UTC	OTC	Probability
*Soil*			
EC_a_, mS m^−1^	11.4 ± 3.5	11.7 ± 3.3	ns
SMC, %			
d-0.10 m	6.4 ± 2.0	7.6 ± 2.1	0.0032
d-0.20 m	9.9 ± 3.5	12.5 ± 2.6	0.0009
d-0.30 m	13.4 ± 6.7	15.7 ± 4.9	0.0011
d-0.40 m	14.7 ± 6.3	17.8 ± 7.3	0.0020
d-0.50 m	18.5 ± 5.4	21.5 ± 7.4	0.0012
d-0.60 m	19.8 ± 5.6	25.0 ± 6.8	0.0006
*Pasture*			
GM, kg ha^−1^	12,402.5 ± 3910.1	21,403.3 ± 9127.9	0.0002
DM, kg ha^−1^	1804.2 ± 545.5	2986.7 ± 1194.5	0.0001
CP, %	13.4 ± 4.2	10.5 ± 1.8	0.0375
PAR, μmol m^−2^ s^−1^	239.7 ± 56.5	1332.5 ± 121.1	0.0000
T_mean_, °C	13.2 ± 0.8	14.9 ± 2.0	0.0108
CMR	6994.1 ± 1198.5	10,133.9 ± 1552.9	0.0000
NDVI	0.741 ± 0.078	0.723 ± 0.050	0.0491

EC_a_—Apparent soil electrical conductivity; SMC—Soil moisture content; d—depth; GM—Green matter; DM—Dry matter; CP—Crude protein; PAR—Photosynthetically active radiation; T_mean_—Mean temperature; CMR—Corrected meter readings of capacitance probe; NDVI—Normalized difference vegetation index; Probability- Probability of significant differences (*p* < 0.05%).

**Table 2:** Suitability of different sensing technologies for characterizing and to managing *montado* ecosystem: comparative analysis based on characteristics and potential applications.

Characteristics and Potential Applications	Proximal Sensing	Remote Sensing UAV-Based	Remote Sensing Satellite-Based
Characteristics			
*Scale*	Low	Medium	High
*Resolution*	High	Medium	Low
*Cost*	High	Medium	Low
Perspective of application			
*Research*	High	High	High
*Business*	Low	Medium	High
Potential to differential management			
*Soil (fertilizer or correction)*	High	Medium	Medium
*Pasture (resowing of botanical species or rotation of grazing plots)*	High	High	High
*Tree (density or diseases)*	Low	High	High
*Animal (feed supplementation/ tracking)*	High/High	High/Low	High/Low
